# Glycan analysis of *Fonsecaea monophora* from clinical and environmental origins reveals different structural profile and human antigenic response

**DOI:** 10.3389/fcimb.2014.00153

**Published:** 2014-10-31

**Authors:** Juliana R. Burjack, Arquimedes P. Santana-Filho, Andrea C. Ruthes, Daniel S. Riter, Vania A. Vicente, Larissa M. Alvarenga, Guilherme L. Sassaki

**Affiliations:** ^1^Química de Carboidratos, Departamento de Patologia Básica, Universidade Federal do ParanáCuritiba, Brazil; ^2^Departamento de Bioquímica e Biologia Molecular, Universidade Federal do ParanáCuritiba, Brazil

**Keywords:** glycan, PCA, dematiaceous fungi, NMR, antigenic response

## Abstract

Dematiaceous fungi constitute a large and heterogeneous group, characterized by having a dark pigment, the dihydroxynaftalen melanin—DHN, inside their cell walls. In nature they are found mainly as soil microbiota or decomposing organic matter, and are spread in tropical and subtropical regions. The fungus *Fonsecaea monophora* causes chromoblastomycosis in humans, and possesses essential mechanisms that may enhance pathogenicity, proliferation and dissemination inside the host. Glycoconjugates confer important properties to these pathogenic microorganisms. In this work, structural characterization of glycan structures present in two different strains of *F. monophora* MMHC82 and FE5p4, from clinical and environmental origins, respectively, was performed. Each one were grown on Minimal Medium (MM) and Czapeck-Dox (CD) medium, and the water soluble cell wall glycoconjugates and exopolysaccharides (EPS) were evaluated by NMR, methylation and principal component analysis (PCA). By combining the methylation and 2D NMR analyses, it was possible to visualize the glycosidic profiles of the complex carbohydrate mixtures. Significant differences were observed in β-D-Gal*f*-(1→5) and (1→6) linkages, α- and β-D-Glc*p*-(1→3), (1→4), and (1→6) units, as well as in α-D-Man*p*. PCA from ^1^H-NMR data showed that MMHC82 from CD medium showed a higher variation in the cell wall carbohydrates, mainly related to O-2 substituted β-D-Gal*f* (δ 106.0/5.23 and δ 105.3/5.23) units. In order to investigate the antigenic response of the glycoconjugates, these were screened against serum from chromoblastomycosis patients. The antigen which contained the cell wall of MMHC82 grown in MM had β-D-Man*p* units that promoted higher antigenic response. The distribution of these fungal species in nature and the knowledge of how cell wall polysaccharides and glycoconjugates structure vary, may contribute to the better understanding and the elucidation of the pathology caused by this fungus.

## Introduction

The family Herpotrichiellaceae contains a large group of fungi whose main characteristic is the presence of melanized hyphae and conidia. Some dematiaceous fungi belonging to this family are pathogenic to vertebrate hosts. Pathogenic black yeasts are part of this group and during a period of their life cycle they show a yeast-like development. These black fungi are able to withstand adverse growth conditions and cause a variety of infections (Vicente et al., 2001, 2008; López-Ribot et al., 2004; Cunha et al., 2010; Sun et al., 2011). Most dematiaceous fungi are present in soil and found as decomposing vegetation-inhabiting saprobes. Secondary host infection is dependent mainly on three factors: host resistance, amount of inoculum and fungal virulence (Alviano et al., 2003). Those factors are influenced by the composition of the culture media (Viccini et al., 2009).

The principal subcutaneous mycoses caused by these fungi are chromoblastomycosis and phaeohyphomycosis. Chromoblastomycosis is a chronic mycotic infection with a slow evolution that affects skin and subcutaneous cell tissue (Esterre and Queiroz-Telles, 2006; Ameen, 2009; Queiroz-Telles et al., 2009; Sharma et al., 2010; Sugiyama et al., 2011). Phaeohyphomycosis was proposed by Ajello (Ajello et al., 1974) as cutaneous and subcutaneous infections, the most frequent ones and the deepest (systemic), acute or chronic infections, caused by a wide variety of dematiaceous fungi (Revankar et al., 2004).

Cell wall structure is vital for all fungi and is the first cell barrier with its surrounding environment, protecting it from environmental stress (Pinto et al., 2008). Polysaccharides are associated with polypeptides, constituting the cell wall glycoproteins, forming the external layers that are involved in different types of interactions with the extracellular environment. Several of these molecules are immunostimulatory compounds with a great potential, such as pathogenicity regulators and determinants for the host immune response (Silva et al., 2008).

Some of these glycan-containing molecules can be recognized by antibodies from patients and can be used to diagnose fungal infections. Proteins and glycoproteins exposed on the external layers are involved in various types of fungal interactions. These glycoproteins are known to influence the immune response (Alviano et al., 2004; López-Ribot et al., 2004; Pinto et al., 2008).

In this context, we evaluated the growth of strains from clinical and environmental origins of the fungus *Fonsecaea monophora* and studied the water-soluble polysaccharides in two different culture media. Principal component analysis (PCA) and immunological assays were performed with the exopolysaccharides (EPS) and soluble cell wall polysaccharides. The differences between these strains could lead to the understanding of the physiological and virulence mechanisms involved regarding both saprobe and pathogenic strains.

## Materials and methods

### Fungal strains

Two strains of *F. monophora*, of clinical and environmental origins, were used: strain MMHC82 (CBS 102248), isolated from patients with chromoblastomycosis lesions, was kindly furnished by the laboratory of Micology of the Universidade Federal do Paraná Clinical Hospital (HC-UFPR/Curitiba-PR, Brazil), and strain FE5p4 (CBS 102225/DH 11584) isolated from decomposing wood of the Centro Nacional de Pesquisas de Florestas (CNPF/EMBRAPA Colombo-PR, Brazil). These samples have already been morphologically and genetically characterized by CBS (Central Bureau Voor Schimmelcutures, Institute of the Royal Netherlands Academy of Arts and Science), (De Hoog et al., 2004; Vicente et al., 2008).

### Growth curves and EPS production

Each strain was grown in Sabouraud medium for 48 h at 37°C, 5 mL of each pre-inoculum with the strain MMHC82 and FE5p4 was inoculated in 250 mL of either Czapeck-Dox (CD) and Minimum Medium (MM), incubated at 37°C and kept at constant agitation for 48 h. An aliquot (19 mL) was removed from each culture and 1 mL was used to measure the sugar consumption. The remaining sample (18 mL) was used for EPS and cell wall polysaccharide isolation. The solution was filtered (Whatman filter paper grade 1) and the supernatant precipitated with 3 volumes of ethanol. The process was monitored every 48 h for 20 days. The growth and EPS production patterns were assembled based on the biomass and EPS (dry weight, obtained after freeze-drying). In addition to the growth curves and the EPS production, measurements were also obtained on the consumption of sucrose in the CD medium and glucose on the MM medium. Glucose was measured based on glucose oxidase method (DiaSys Diagnostic Systems GMBH & Co.KG), which forms gluconic acid and H_2_O_2_. The latter reacts with the phenol and 4-amineantipyrine, giving rise to p-benzoquinone-monoimine antipyrine (red color) and the concentration of residual glucose determined, in mg/ml, by the absorbance measured at 500 nm (Viccini et al., 2009). Sucrose consumption was determined by Silica-Gel 60G TLC plates (Merck), with sucrose solutions in different concentrations as standard (2.5–10 mg.ml^−1^). Plates were developed with AcOEt:*n*-PrOH:AcOH:H_2_O (4:2:2:1, v/v), and detection of carbohydrates was performed with orcinol-H_2_SO_4_ treatment (Sassaki et al., 2011). The plates were analyzed by densitometry using the Scion Image Program (Scion Corporation, Maryland, USA) and the results plotted in Microsoft Excel 2007 (Sassaki et al., 2005).

### EPS and mycelium polysaccharide extraction

Cell free medium was obtained after centrifugation at 12.000 × g for 20 min and the supernatant was submitted to cold ethanolic precipitation (3:1, v/v) and left overnight at −20°C. The latter gave yield to the native exopolysaccharide, which was centrifuged at 8.400 × g for 10 min. EPS purification was conducted after dialyzed against tap water using membranes with 16 kDa cutoff for 48 h, followed by lyophilization to measure the EPS dry weight production. Mycelia polysaccharides were obtained by aqueous extraction conducted in an autoclave under a pressure of 1 atm at 120°C for 40 min, which assures fungi inactivation and extraction of water-soluble polysaccharides in one step (Farres et al., 1996). The polysaccharides were obtained after precipitation with ethanol (3:1, v/v) of the supernatant solution resulted after removing the mycelia by centrifugation.

### NMR spectroscopy

NMR spectra were obtained with a Bruker 400 MHz AVANCE *III* NMR spectrometer with a 5 mm inverse Z gradient probe. The samples were deuterium exchanged by repeated dissolution in D_2_O and freeze-drying, finally the samples were dissolved in D_2_O solution containing Na^0^ 1 mM. ^1^H-NMR chemical shifts of signals are expressed in δ (ppm), relative to trimethylsilyl propionate sodium salt TMSP (δ = 0) at 70°C. Spectra were acquired using 256 scans to give a S/N ratio of 300 (90° pulse, relaxation delay = 4.0 s, number of time domain points = 65476 and acquisition time = 7.7 s). Integration of H-1 area was performed without tube rotation and respecting a HDO signal with a medium half line width varying from 2.5 to 3.5 Hz after Lorentzian deconvolution and post Fourier transformation. 2D NMR experiment was carried out using HSQC, heteronuclear correlation via double inept transfer with decoupling during acquisition, using trim pulses in inept transfer (hsqcetgpsi) program recorded for quadrature detection in the indirect dimension and acquired using 32 scans per series of 2 K × 400 data points, with zero filling in F1 (4 K) prior to Fourier transformation (Sassaki et al., 2011).

### Methylation analysis

Per-*O*-methylation of each isolated polysaccharide fraction (10 mg each) was carried out using NaOH-Me_2_SO-MeI (Ciucanu and Kerek, 1984). After 30 min at 25°C with vigorous stirring, the mixture was maintained overnight at 25°C. The reaction was interrupted by the addition of water, neutralized with AcOH, dialyzed against distilled water and freeze-dried. The products were submitted to one more cycle of methylation, followed by partition between CHCl_3_ and water. The per-*O*-methylated derivatives were hydrolyzed with 50% v/v aq. H_2_SO_4_ (0.5 ml v/v, 1 h, 0°C), followed by a dilution until it reached 5.5% (addition of 4.0 ml of distilled H_2_O). The solution was kept at 100°C for 12 h and was then neutralized with BaCO_3_, filtered and the filtrate evaporated to dryness. The resulting mixture of *O*-methylaldoses was reduced with NaBD_4_ and acetylated with Ac_2_O-pyridine to give a mixture of partially *O*-methylated alditol acetates (PMAAs) which were analyzed on a GC-MS Varian Saturn 2000R using a DB-225 capillary column (30 m × 0.25 mm i.d.), held at 50°C during injection and then programmed at 40°C.min^−1^ to 215°C (constant temperature). PMAAs electron impact (EI) spectra were obtained at 70 eV at 200°C, and post-run analysis was performed with a Saturn Workstation 5.1, the identification of PMAAs being confirmed using selected PMAAs mixtures, according to Sassaki et al. (2005).

### Monosaccharide analysis

Cell-wall glycoconjugates (0.1 mg) were hydrolyzed with 2 M trifluoroacetic acid (1 mL) at 100°C for 8 h. After evaporating the acid, the resulting monosaccharides were converted to their alditol acetates through successive NaBH_4_ reduction and acetylation with Ac_2_O-pyridine and then analyzed by GC-MS. Monosaccharide analyses were performed using a Varian 4000 MS equipped with 30 m × 0.25 mm low bleed/MS capillary columns. The alditol-acetates were submitted to GC-MS analysis using capillary columns of CP-Sil-5CB programmed as follows: injector 250°C, oven start at 50°C (hold 2 min) to 90°C (20°C.min^−1^, then held for 1 min), 280°C (5°C.min^−1^, then held for 2 min) and to 310°C (3°C.min^−1^, then held for 5 min) (Sassaki et al., 2008). EI spectra were obtained at 70 eV at 200°C. Post-run analysis was performed with a Saturn Workstation.

### Principal component analysis

Chemometric analysis, chemometrics or multivariate analysis is a valuable mathematical statistics tool, which along with different analytical techniques allows several variables to be analyzed in a single sample. In many cases, the visual inspection of NMR data reveals only a small amount of information. Thus, statistical methods have been used to extract the maximum of information from these data.

The principal component analysis (PCA) is the most widely used method among multivariate techniques. PCA is an additive linear method in the sense that each principal compound has a share of importance in the data set. Generally, a small quantity of principal components totalizes over 90% of the total variance, and in these cases, data can be resized in small PCs, thus reducing the dataset dimension.

PCA analysis from NMR data was performed on AMIX v 3.8.3 (Bruker) and input variables were obtained directly from the raw NMR data. The buckets were built using a width of 0.01 ppm, ranging from 5.50 to 4.70 ppm. The method of integration was scaling in relation to the total intensity of signals.

### Human serum

For the immunological assays 12 sera samples obtained from chromoblastomycosis patients were used. Negative control was performed in parallel using six sera samples from disease-free individuals. All samples were furnished by the Laboratory of Micology of the HC-UFPR. The use of all biological material obtained from patients was approved by the Human Research Ethics Committee of the HC-UFPR, registry number 2126.021/2010-01. The inclusion criteria used by the serum bank of the Clinical Hospital was the confirmation for the diagnosis of chromoblastomycosis by performing direct mycological, culture and/or histopathological examination. The exclusion criteria used were negative results of the above examinations.

### Antibody production and evaluation

Sera samples from chromoblastomycosis patients were evaluated in regard to their capacity to form immune complexes by the reaction of each serum antibody and glycoconjugates obtained from fungal cell wall of *F. monophora* strains. ELISA tests were performed with the glycoconjugate antigens present in *F. monophora*. 96-well plates were coated with 100 μL of a 10 μg.ml^−1^ carbohydrate-containing antigens for 12–16 h at 4°C. After the coating period, the polystyrene plate was rinsed thrice with a wash buffer (0.05% Tween saline) and blocked with a 2% casein solution diluted in PBS for 2 h at 37°C. The plate was rinsed again as described above and incubated with sera from the patients, diluted into incubation buffer (0.25% casein diluted in PBS, 0.05% Tween 20, Bio-Rad, Hercules, CA). As a negative control, sera samples from disease-free individuals were used. The second antibody added to the plate consisted of anti-human IgG immune Fc specific immunoglobulins with peroxidase (Sigma, St. Louis, MO) diluted 1:1000 into incubation buffer. The enzymatic activity was evaluated using ortophenilen-diamine (OPD) solution; after 15 min of incubation, the reaction was halted by the addition of 20 μL of diluted sulfuric acid (1:20) and the absorbance was measured at 490 nm.

## Results and discussion

### Growth evaluation and EPS production for FE5p4 and MMHC82 strains

**Figure 1:**
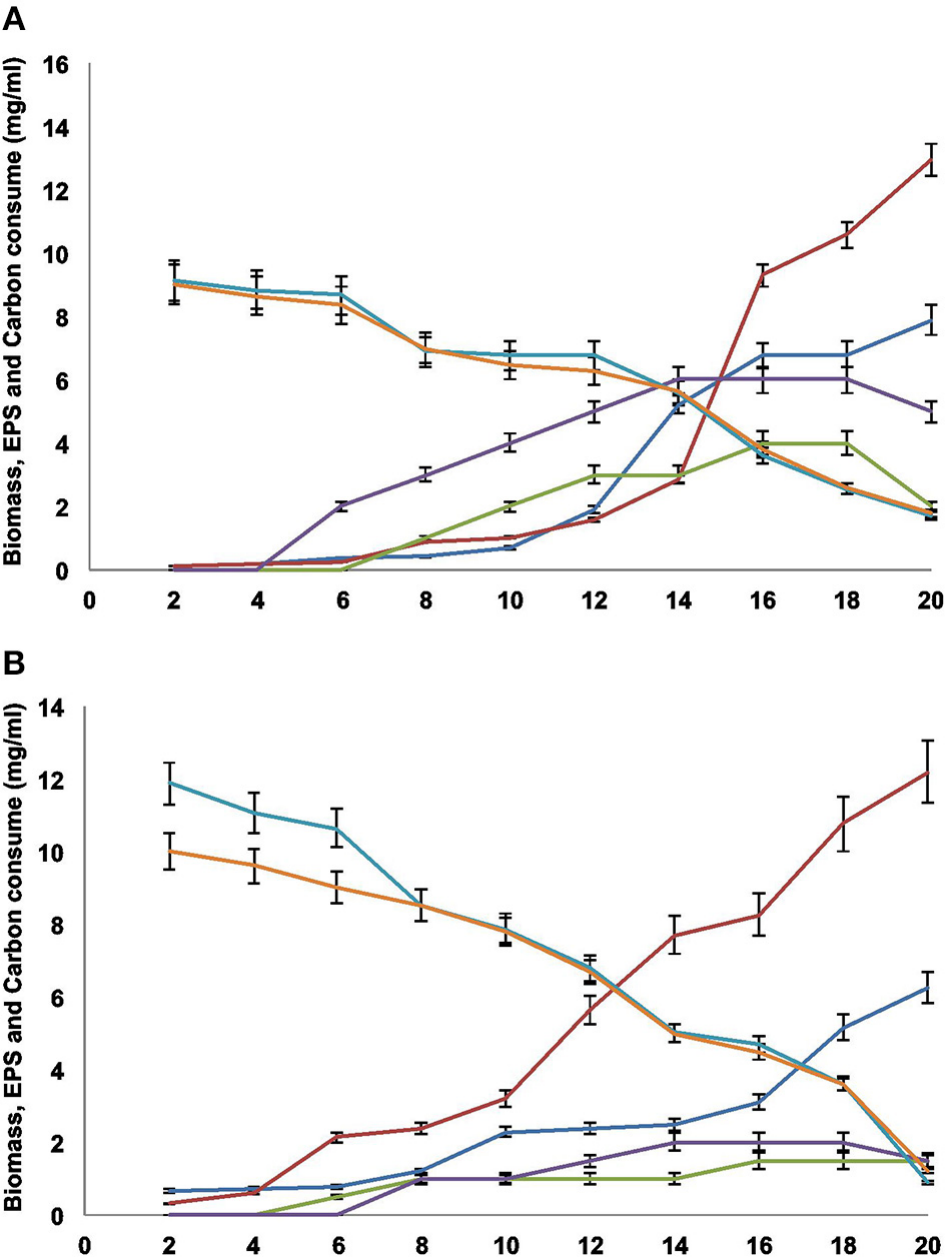
**(A)** FE5p4 and **(B)** MMHC82. Profiles for (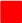) biomass MM, (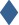) biomass CD, (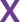) EPS MM, (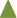) EPS CD, (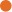) sucrose consumption and (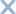) glucose consumption. The results are expressed as the average of triplicates±SE.

Biomass increased significantly after the eighth day of culture, and the best medium to obtain biomass, for both FE5p4 and MMHC82 strains, was MM (Figures 1A,B, respectively). It was observed that the addition of an organic substrate to the medium (sucrose or glucose), increases biomass proportionally, as well as EPS production, mainly after the fourth day.

Also, glucose levels on MM decreased throughout the 20 days of culture (Figures 1A,B), and was nearly depleted at the twentieth day. In CD medium, sucrose consumption was also assessed, and similarly decreased throughout the 20 days. The lack of a carbon source in the medium may explain the decrease in the amount of EPS recovered from the cultures (Figures 1A,B), probably due to the use of the polysaccharide as a carbon source.

A higher production of EPS was obtained for MM cultures when compared to CD. According to Silva and coworkers (Silva et al., 2006), the carbon source can determine the amount of polysaccharides formed, as well as the glycosidic composition of the synthesized polymer.

Since the sugar sources did not deplete completely, as shown on Figures 1A,B, the EPS production was maintained for both strains during their exponential growth phases. The highest amounts were found in the sixteenth, seventeenth, and eighteenth days. However, after the twentieth day, EPS production decreased significantly, probably due to degradation by the fungi and the use of EPS as carbon source in response to low sugar levels in the media, as discussed previously. This physiological behavior may be described as a survival strategy for these microorganisms, since EPS may act as an energy reserve during cellular stress. As reported by Yurlova and de Hoog (Yurlova and De Hoog, 2002; De Hoog et al., 2004), differences on EPS production using different culture media were found, however timed data was only acquired by the end of the fifth day of culture, not throughout the entire growth period.

### Monosaccharide composition of EPS and cell wall polysaccharides

The EPS obtained from both strains showed high amounts of mannose, galactose, and glucose either for MM and CD cultures. The main differences observed were the higher relative amount of galactose found for strain MMHC82 in comparison to glucose when growth on both CD and MM medium. The EPS produced by strain Fe5p4 showed a higher amount of glucose when cultivated on CD medium compared to MM medium. For both strains, independent of growth condition, the most abundant monosaccharide found on EPS composition was mannose.

In previous studies performed by Alviano et al. (1991, 2003), which evaluated melanin and carbohydrate composition of *F. monophora* exopolysaccharides, similar results were found. According to Barreto-Bergter et al. (2008) carbohydrates from *Scedosporium prolificans*, an opportunist pathogen that causes a localized infection in tissues and bones of immunocompromised patients, were compared to those from *Pseudallescheria boydii* and they observed as primary monosaccharides mannose and rhamnose, followed by galactose and glucose (Barreto-Bergter et al., 2008)

Cell wall polysaccharides produced by fungal strains used in this work, grown on both MM and CD medium, had a higher relative amount of glucose when compared to mannose and galactose (Table 1 in Supplementary Material). A similar composition has been previously reported for the cell wall of other species of pathogenic fungi, such as *Histoplasma capsulatum* (Gorocica et al., 2009). However, for *Aspergillus fumigatus, A. wentii* and *Chaetosartory chrysella*, mannose-containing molecules were also found forming cell walls as galactomannans with distinct chemical and physical properties (Gomez-Miranda et al., 2003).

### Nuclear magnetic resonance and methylation analysis of the cell wall polysaccharides

Besides the determination of the monosaccharide composition, methylation and NMR analyses were performed in order to determine the structural characteristics of the cell wall polysaccharides obtained from both strains grown on MM and CD cultures (Table 2 in Supplementary Material, Figures 2A–D).

**Figure 2:**
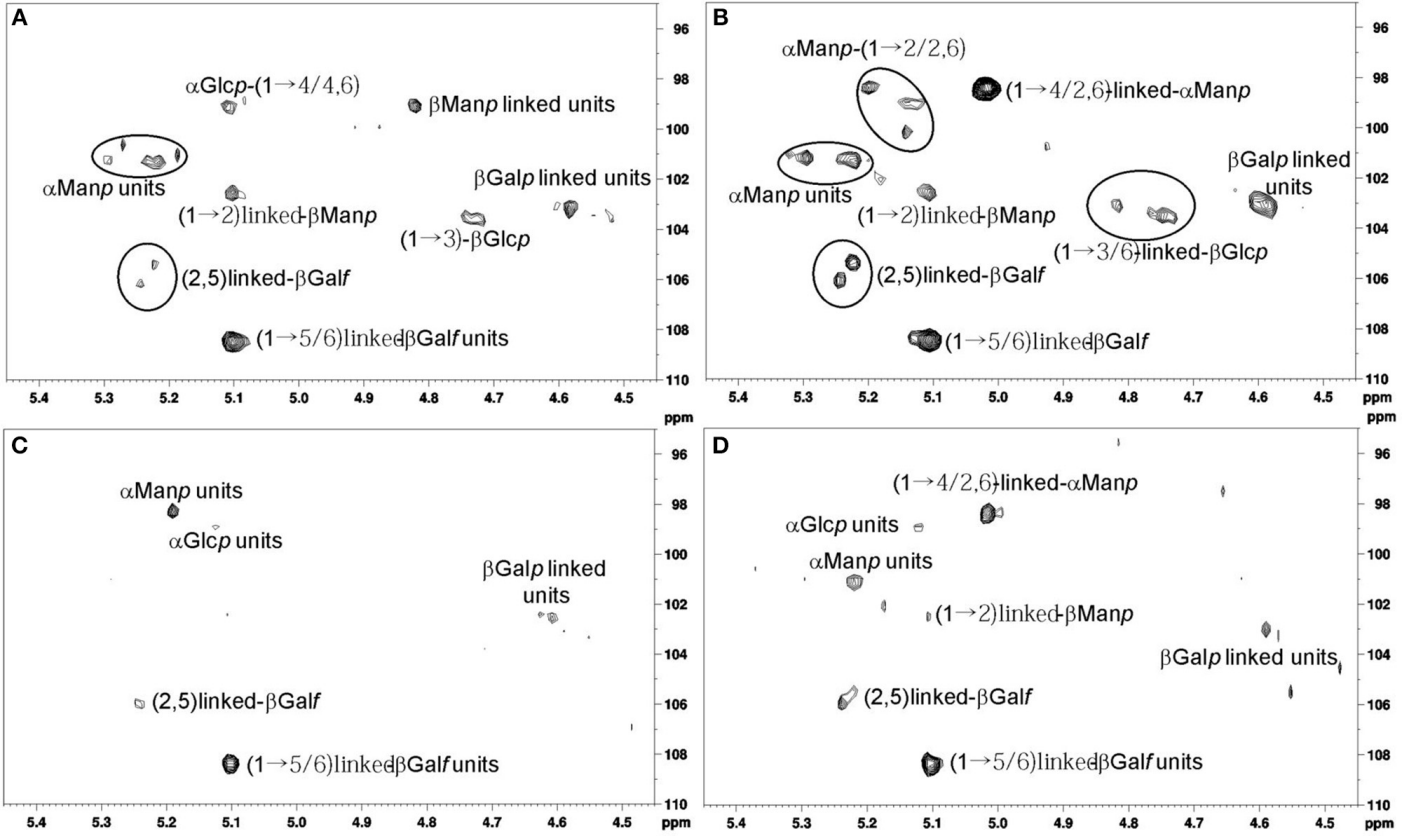
**(A)** Anomeric region analysis of strain MMHC82 spectrum for MM. **(B)** Anomeric region analysis of strain MMHC82 spectrum for CD medium. **(C)** Anomeric region analysis of strain FE5p4 spectrum for MM. **(D)** Anomeric region analysis of strain FE5p4 spectrum for CD medium.

The anomeric region for the polysaccharide of strain MMHC82 grown on CD medium showed characteristic signals with chemical shifts attributed to α-linked monosaccharides at δ 101.3/5.21 and δ 98.4/5.01 as well as β-linked units at δ 108.4/5.10, δ 106.0/5.23, δ 105.3/5.21, δ 103.5/4.73, and δ 103.0/4.50 (Figure 2A) (Gorin and Mazurek, 1975). Similar signals were found for this strain when grown on MM, however with different relative intensities among the peaks (Figure 2B).

The main difference found for this strain when comparing growth condition is the presence of signals corresponding to β-D-Gal*f* units (δ 106.0/5.23 and δ 105.3/5.23), on CD medium, in contrast to the signals attributed to α-D-Man*p* (δ 99.1/5.11) and β-D-Man*p* (δ 99.1/4.82 and 101.3/5.11) (Viccini et al., 2009) found for the MM samples (Figures 2A,B).

The spectrum obtained for strain FE5p4 grown in CD medium had H1/C1 cross peaks corresponding to α-anomeric configuration at δ 101.1/5.22 and δ 98.4/5.01, and also signals attributed to β-linked units at δ 108.3/5.10, δ 105.9/5.23, and δ 103.0/4.59, which shows a similar profile to the spectrum obtained for strain MMHC82, also with great differences on the peaks relative intensities (Figure 2C). Examination of the 2D NMR spectrum profile for MM-grown samples showed major differences especially regarding the presence of anomeric signals attributed to α-D-Man*p* units, at δ 101.1/5.22 (Figures 2A,C) (Barreto-Bergter et al., 2008). On CD medium, the differentiation of the strains were related to α-D-Man*p* (1→6) and β-D-Man*p* (1→2) (Figures 2B,D), (Viccini et al., 2009).

Biomolecules containing D-Gal*f* residues have been described as important antigens among several human pathogenic fungi. Since Gal*f* is not found in human hosts, these molecules could be a differential antigen between fungus and host, allowing cytokine induction and immunological activation. Monoclonal antibodies tested against these structures have shown to be effective on the detection of this type of antigen. The same has been reported for biomolecules containing D-Man*p* units, since it can stimulate antibody production and T-cell activation thus preventing the development of the infection process by this fungus (Stynen et al., 1992).

Bittencourt et al. (2006) characterized the α-D-glucan structure of a polysaccharide found on the cell wall of *Pseudallescheria boydii*, and assessed its role in the innate immune response, stating that the lack of such structures diminishes phagocytosis. This suggests that α-D-glucan plays an important role in macrophage and dendritic cell stimulation for the immune system. It has also been reported that pathogenicity in fungi is dependent on β-D-glucans (Stahl and Ezekowitz, 1998).

Methylation analysis of the cell wall polysaccharides obtained from the strain FE5p4 showed a backbone containing D-Man*p* (Barreto-Bergter et al., 2008; Gorin et al., 2010) and D-Gal*p* residues, both C6-linked (Leal et al., 2008), with branching units of D-Man*p*, D-Ga*lf*, and D-Glc*p* at O-3. For the same strain grown on MM medium, the cell wall polysaccharides showed similar structural characteristics, with backbone units being 3-*O*-substituted by D-Man*p*, D-Gal*f*, and D-Glc*p* in different ratios.

Strain MMHC82 produced a mannan as the main cell wall component, with a backbone predominantly composed of D-Man*p* (1→2) and D-Man*p* (1→6)-linked when grown on CD medium, and D-Man*p* (1→2) and D-Man*p* (1→4)-linked on MM culture. Regarding the 3-*O*-substitutions in D-Man*p*, D-Gal*f*, and D-Glc*p* units were most present in the cell wall polysaccharide produced by this strain on either medium, with 6-*O*- substitutions appearing for the MM condition.

### Chemometric analysis

**Figure 3:**
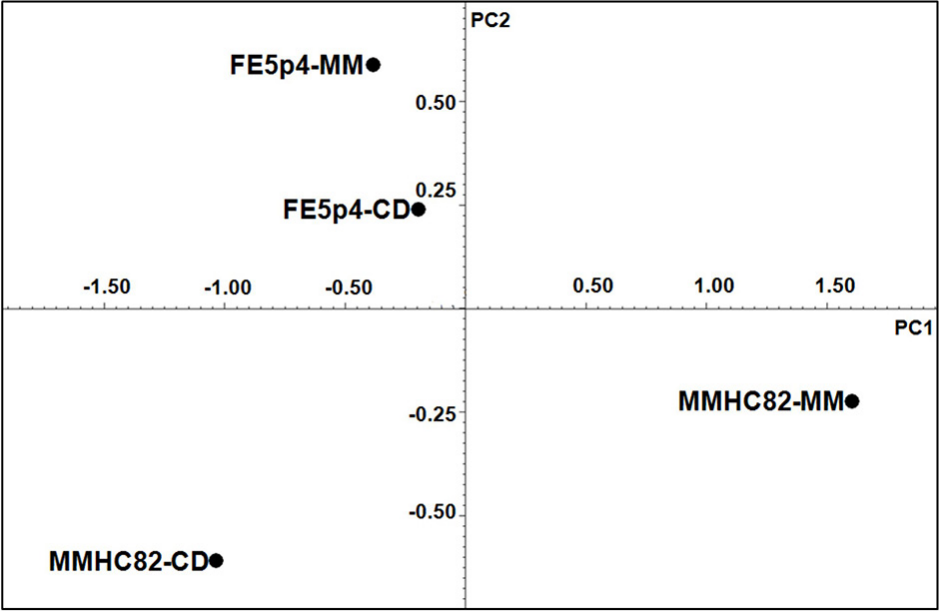
**Chemometric analysis from ^1^H NMR spectra**.

Principal component analysis (PCA) was performed using the acquired NMR data from cell wall samples. PCA results suggest that the culture conditions promote differentiation of the polysaccharide structures on both environmental and pathogenic strains. PC1 of the NMR data explains approximately 81.6% of the total variance and PC2 explain 17.5% of the variance (Figure 3).

The ^1^H-NMR results showed that both strains, when grown on MM medium, shows a significant structural variability on cell wall polysaccharide structures. As shown on Figure 3, strain FE5p4 (non-pathogenic), seems to be related to the pathogenic strain when the water soluble cell wall polysaccharides, identified from growth on CD, are compared, suggesting that MM medium is more suitable to show the differences between the polysaccharide synthesized by each strain.

### Antibodies evaluation

ELISA testing was performed using sera from patients with chromoblastomycosis, as well as from disease-free individuals as control to check for reactivity against the water-soluble cell wall polysaccharides (antigens) of strains MMHC82 and FE5p4 of the fungus *F. monophora*.

**Figure 4:**
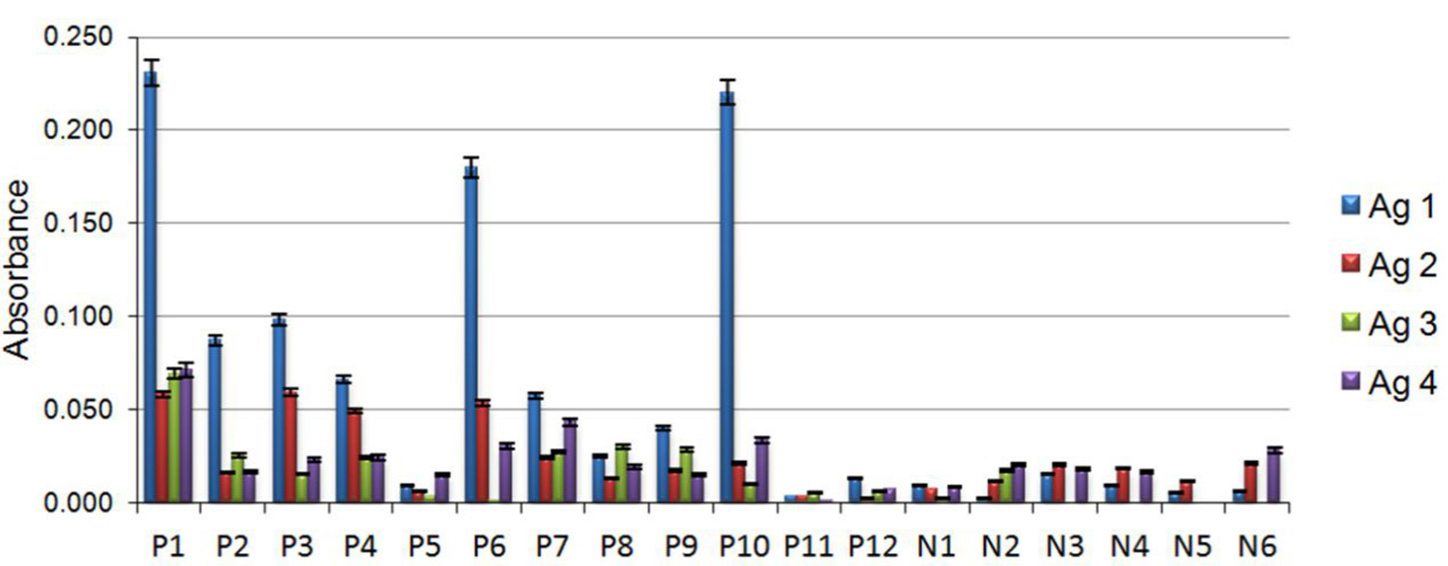
**Individual data from immunoassays with antigens existing on the serum of patients with chromoblastomycosis (P), patients without chromoblastomycosis (N)**. Antigens were assessed to determine their capacity of reaction in the formation of immune complexes with wall polysaccharides of the fungus *F. monophora*. Antigen 1 (Ag1), belonging to the cell wall of strain MMHC82 drawn from MM, antigen 2 (Ag2), cell wall antigen of strain MMHC82 from CD medium, antigen 3 (Ag3), cell wall antigen from strain FE5p4 from MM and antigen 4 (Ag4), cell wall antigen from strain FE5p4 from CD medium.

Antigens obtained from both strains grown on each condition were denominated as follows: Antigen 1 (Ag1), obtained from strain MMHC82 grown on MM; Antigen 2 (Ag2), from strain MMHC82 on CD medium; Antigen 3 (Ag3), from strain FE5p4 grown on MM; and Antigen 4 (Ag4), from strain FE5p4 on CD medium (Figure 4 and Figure S1).

The results for the antigen-antibody reaction using sera from chromoblastomycosis patients showed a great significance. Polysaccharide antigen Ag1 showed the best antigenic response to sera from patients with chromoblastomycosis, giving rise to a 91% increase on absorbance when compared to results from the control group. Ag2, Ag3, and Ag4 were shown to be much less reactive to the antibodies present in the sera of infected patients, and no significant difference was observed on absorbance in comparison to the non-chromoblastomycosis control groups. The majority of sera samples obtained from patients showed markedly reactivity against the antigens, particularly against Ag1, although there were some sera from patients that showed low reactivity to all the antigens (P5, P11, and P12), which can be a consequence of many factors, such as geographic prevalence of the fungal strains, antigenic variation and individual-specific differences in immune response (Vera-Cabrera et al., 2012; Wevers et al., 2014).

## Conclusions

When grown on MM and CD medium, the fungus *F. monophora* produces an EPS containing mannose, galactose and glucose in variable proportions depending on the strain. MM has shown to be the best of the tested conditions to produce these polymers and also greater amount of biomass.

Water soluble cell wall polysaccharides from both strains also had glucose, mannose and galactose in their composition. Methylation analysis and NMR of the polysaccharides showed a backbone with D-Man (1→6) and D-Gal*f* (1→6) units with 3-*O*-substitutions, indicating the presence of a galactofuranan, together with the presence of a (1→4)-linked α-D-glucan branched at O-6, a “glycogen-like” polysaccharide, a β-D-glucan (1→3), (1→6)-linked and also the presence of α-D-mannans (1→6) substituted at O-2 and O-4.

Chemometric analysis of the ^1^H-NMR data showed that strain MMHC82 growth in MM medium, had great variation in its biochemical profile regarding cell wall polysaccharides, discriminating the clinical from the environmental strain as observed in PC1 which explains 81.6% of the total variance.

The antigen from water-soluble cell wall polysaccharide obtained from the strain MMHC82 (pathogenic) when grown on MM was the only sample that showed high antigenic response against the antibodies found in the sera from patients with chromoblastomycosis. All other cell wall polysaccharides tested were undifferentiated from the control experiment. These results were attributed to the presence of β-D-Man*p* in Ag1 which is not found in the other antigen samples, as supported by NMR and methylation analysis.

The results presented in this manuscript highlight the importance to study macromolecules from clinical and environmental fungal strains. The PCA results proved useful, as they could discriminate between strains with different ecological characteristics using the data obtained from ^1^H-NMR analysis. Another important conclusion of this study was to show that the β-D-Man*p* seems to play an important role on the antigenic response. The structural diversity of the polysaccharides found for both strains using two media, supports the use of these macromolecules as physiological fingerprints, aiming toward both taxonomic identification and antigenic studies.

### Conflict of interest statement

The Associate Editor Eliana Batteto Bergter declares that, despite having collaborated with author Guilherme Sassaki, the review process was handled objectively. The authors declare that the research was conducted in the absence of any commercial or financial relationships that could be construed as a potential conflict of interest.

## Supplementary material

Figure S1 **Immunoassay with antigens existing on the serum of patients with chromoblastomycosis (black bars), patients without chromoblastomycosis (white bars)**. Antigens were assessed to determine their capacity of reaction in the formation of immune complexes with wall polysaccharides of the fungus *F. monophora*. Antigen 1 (Ag1), belonging to the cell wall of strain MMHC82 drawn from MM, antigen 2 (Ag2), cell wall antigen of strain MMHC82 from CD medium, antigen 3 (Ag3), cell wall antigen from strain FE5p4 from MM and antigen 4 (Ag4), cell wall antigen from strain FE5p4 from CD medium. Results correspond to the medium ± E.P.M of 6–12 samples. ^***^*p* < 0.001 in relation to the control group, serum of non-chromoblastomycosis patient (ANOVA of a pathway followed by *t*-test with a Bonferroni adjustment).
