# Supplementary material for: Glycan analysis of Fonsecaea monophora from clinical and environmental origins reveals different structural profile and human antigenic response
Source: Front Cell Infect Microbiol. 2014 Oct 31;4:153. doi: 10.3389/fcimb.2014.00153 (PMC4215789; doi:10.3389/fcimb.2014.00153)
Supplement: Supplementary file 1 [file Table1.DOC]

**Table 1.** Monosaccharide composition of EPS and cell wall polysaccharides of strains MMHC82 and FE5p4 obtained during growth on media CD and MM.

|  |  |  |  |  |  |  |  |  |
| --- | --- | --- | --- | --- | --- | --- | --- | --- |
|  | MM Medium | | | | | | | |
|  | **Rha** | **Fuc** | **Rib** | **Ara** | **Xyl** | **Man** | **Gal** | **Glc** |
| EPS MMHC82 | 0.8 | 3.6 | 2.8 | 0.3 | 1.0 | 53 | 25.2 | 12.9 |
| EPS FE5p4 | 0.2 | 2.9 | 0.6 | 0.4 | 2.6 | 45.1 | 32.7 | 14.9 |
| Cell Wall MMHC82 | 1.0 | 8.9 | 1.6 | 2.1 | 10.5 | 24.1 | 10.2 | 41.3 |
| Cell Wall FE5p4 | 3.5 | 12.6 | 4.8 | 1.0 | 0.6 | 16.6 | 10.0 | 50.4 |
|  | CD Medium | | | | | | | |
| EPS MMHC82 | 9.4 | 12.0 | 3.1 | 0.8 | 0.7 | 35.9 | 21.7 | 16.0 |
| EPS FE5p4 | 2.7 | 4.7 | 1.3 | 1.2 | 1.6 | 43.9 | 19.6 | 24.6 |
| Cell Wall MMHC82 | 0.9 | 8.1 | 2.2 | 1.1 | 3.4 | 23.2 | 22.2 | 38.6 |
| Cell Wall FE5p4 | 1.0 | 15.3 | 0.4 | 0.7 | 1.9 | 27.0 | 18.7 | 34.7 |
|  |  |  |  |  |  |  |  |  |
