# Supplementary material for: Glycan analysis of Fonsecaea monophora from clinical and environmental origins reveals different structural profile and human antigenic response
Source: Front Cell Infect Microbiol. 2014 Oct 31;4:153. doi: 10.3389/fcimb.2014.00153 (PMC4215789; doi:10.3389/fcimb.2014.00153)
Supplement: Supplementary file 2 [file Table2.DOC]

**Table 2.** *O*-Me*-* alditol acetates obtained by methylation analysed by GC-EM (column DB-225).

|  |  |  |  |  |  |
| --- | --- | --- | --- | --- | --- |
| **Partially methylated alditol-acetates^a^** | **Fraction (%) ^b^** | | | | **Linkages ^c^** |
|  | **MMHC82 CD** | **MMHC82 MM** | **FE5p4 CD** | **FE5p4** |  |
|  |  |  |  | **MM** |  |
| 3,4-Me_2_-Fuc*p* | 8.4 | 6.4 | 3.9 | 9 | →2)-Fuc*p-*(1→ |
| 2,3,4,6-Me_4_-Man*p* | 9.6 | 10 | 10.3 | 11.2 | Man*p*-(1→ |
| 2,3,4,6-Me_4_-Glc*p* | 8 | 7.2 | 7.1 | 10.9 | Glc*p*(1→ |
| 2,3,5,6-Me_4_-Gal*f* | 7.8 | 11 | 15.6 | 14.9 | Gal*f-*(1→ |
| 2,3,4,6-Me_4_-Gal*p* | 4.3 | 4 | 4.5 | 6 | Gal*p*-(1→ |
| 3,4,6-Me_3_-Man*p* | 19.4 | 25 | - | - | →2)-Man*p-*(1→ |
| 2,4,6-Me_3_-Glc*p* | 4 | 4.9 | 3.8 | 10.8 | →3)-Glc*p-*(1→ |
| 2,3,6-Me_3_-Man*p* | 3.1 | 9.5 | 5.4 | - | →4)-Man*p-*(1→ |
| 2,3,4-Me_3_-Glcp | 2.8 | 3.4 | 6.6 | 4.1 | →6)-Glc*p-*(1→ |
| 2,3,4-Me_3_-Man*p* | 20.7 | 4.1 | 20.6 | 14.7 | →6)-Man*p-*(1→ |
| 2,3,5-Me_3_-Gal*f* | 5 | 4.2 | 10.3 | 7.6 | →6)-Gal*f-*(1→ |
| 2,3,4-Me_3_-Gal*p* | 1.9 | - | - | - | →6)-Gal*p-*(1→ |
| 2,3-Me_2_-Man*p* | 0.5 | - | - | - | →4,6)-Man*p-*(1→ |
| 2,3-Me_2_-Gal*f* | 0.7 | - | 1.9 | - | →5,6)-Gal*f-*(1→ |
| 2,4-Me_2_-Gal*p* | 3.8 | 4.2 | 1.7 | 4.2 | →3,6)-Gal*p-*(1→ |
| 3,4-Me_2_-Man*p* | - | 6.1 | - | - | →2,6)-Man*p-*(1→ |
| 2,4-Me_2_-Man*p* | - | - | 8.3 | 6.6 | →3,6)-Man*p-*(1→ |
|  | | | | | |

^a^ *O*-Me*-* alditol acetates obtained from methylation, followed by acid hydrolysis (Sulfuric acid 72%, 100ºC for 8h), reduction with NaB_2_H_4_ and acetylation, analyzed by GC-MS (column DB-225).

^b^ Relative percentage to the areas of all peaks.

^c^ Based on the *O*-methyl alditol acetate derivative
