# Supplementary figures and images for: Glycan analysis of Fonsecaea monophora from clinical and environmental origins reveals different structural profile and human antigenic response
Source: Front Cell Infect Microbiol. 2014 Oct 31;4:153. doi: 10.3389/fcimb.2014.00153 (PMC4215789; doi:10.3389/fcimb.2014.00153)

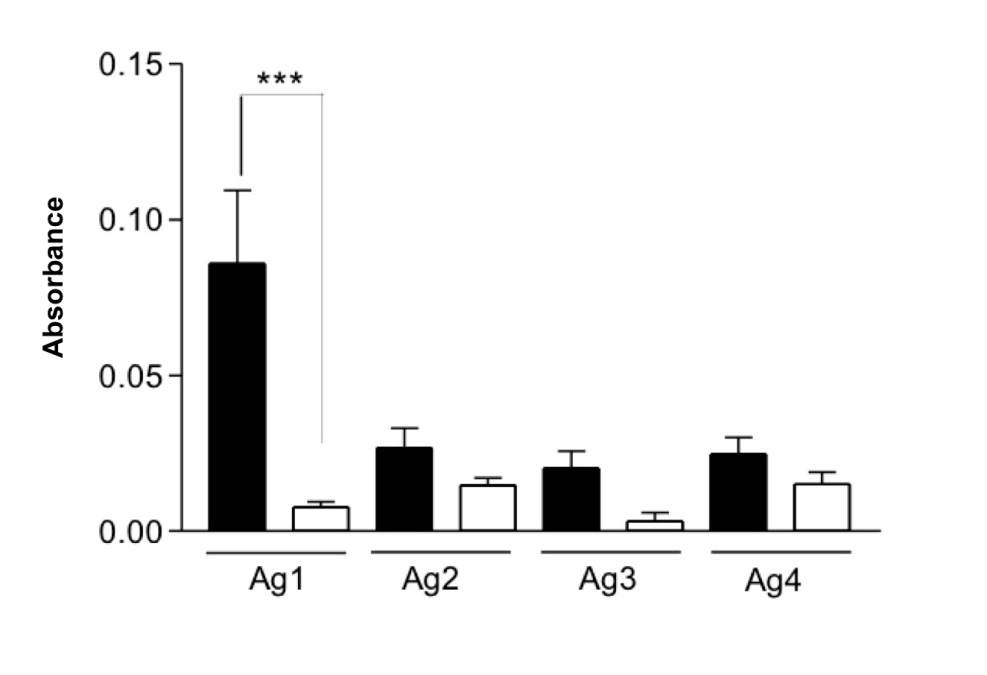

Supplement: Figure S1 — Immunoassay with antigens existing on the serum of patients with chromoblastomycosis (black bars), patients without chromoblastomycosis (white bars). Antigens were assessed to determine their capacity of reaction in the formation of immune complexes with wall polysaccharides of the fungus F. monophora. Antigen 1 (Ag1), belonging to the cell wall of strain MMHC82 drawn from MM, antigen 2 (Ag2), cell wall antigen of strain MMHC82 from CD medium, antigen 3 (Ag3), cell wall antigen from strain FE5p4 from MM and antigen 4 (Ag4), cell wall antigen from strain FE5p4 from CD medium. Results correspond to the medium ± E.P.M of 6–12 samples. ***p < 0.001 in relation to the control group, serum of non-chromoblastomycosis patient (ANOVA of a pathway followed by t-test with a Bonferroni adjustment). [file Image1.JPEG]
